# Constitutive aneuploidy and genomic instability in the single‐celled eukaryote *Giardia intestinalis*

**DOI:** 10.1002/mbo3.351

**Published:** 2016-03-23

**Authors:** Pavla Tůmová, Magdalena Uzlíková, Tomáš Jurczyk, Eva Nohýnková

**Affiliations:** ^1^ Department of Tropical MedicineFirst Faculty of Medicine, Charles University in PragueStudnickova 7Praha 212800Czech Republic; ^2^ Department of Probability and Mathematical StatisticsFaculty of Mathematics and Physics, Charles University in PraguePraha 2Czech Republic

**Keywords:** Aneuploidy, chromosome, FISH, giardia, karyotype, protist

## Abstract

*Giardia intestinalis* is an important single‐celled human pathogen. Interestingly, this organism has two equal‐sized transcriptionally active nuclei, each considered diploid. By evaluating condensed chromosome numbers and visualizing homologous chromosomes by fluorescent *in situ* hybridization, we determined that the *Giardia* cells are constitutively aneuploid. We observed karyotype inter‐and intra‐population heterogeneity in eight cell lines from two clinical isolates, suggesting constant karyotype evolution during in vitro cultivation. High levels of chromosomal instability and frequent mitotic missegregations observed in four cell lines correlated with a proliferative disadvantage and growth retardation. Other cell lines, although derived from the same clinical isolate, revealed a stable yet aneuploid karyotype. We suggest that both chromatid missegregations and structural rearrangements contribute to shaping the *Giardia* genome, leading to whole‐chromosome aneuploidy, unequal gene distribution, and a genomic divergence of the two nuclei within one cell. Aneuploidy in *Giardia* is further propagated without p53‐mediated cell cycle arrest and might have been a key mechanism in generating the genetic diversity of this human pathogen.

## Introduction

The accurate propagation of genomic information to subsequent generations is crucial for the efficient proliferation of any living organism. Alteration through different mechanisms leads to chromosomal rearrangements or whole‐chromosome aneuploidy, which usually has a detrimental impact on an individual′s health, viability, and reproduction (Torres et al. 2008). Aneuploidy is tightly associated with cancer development as either a driver or a passenger in the transformation process (Gordon et al. 2012). Interestingly, in some pathogenic eukaryotic microbes, aneuploidy is constitutive with no significant limitations to cell fitness. Aneuploidy is a source of a genetic heterogeneity and is hypothesized to be beneficial for adaptability, virulence, and drug resistance within the parasite–host interface (Selmecki et al. 2006; Polakova et al. 2009; Sionov et al. 2010). Among single‐celled eukaryotes, aneuploidy is widespread and well‐investigated in environmental, industrial, and pathogenic fungi (Morrow and Fraser 2013; Forche 2014). Aneuploid karyotypes were also found in few examples of parasitic flagellates from the Kinetoplastida group (Minning et al. 2011; Sterkers et al. 2011). Here, we present another eukaryotic microbe with constitutive aneuploidy and describe the impact of the stability of karyotype on the proliferation features of this species.

The single‐celled flagellated parasite *Giardia intestinalis* (Diplomonads, Excavata) is an evolutionarily distant binucleated eukaryote and an important human and veterinary pathogen. The coexistence of two same‐sized transcriptionally active nuclei within a *Giardia* cell provides additional complexity to the karyotype organization of this organism. The transcriptional interplay and coordinated cell cycle progression of the two nuclei is puzzling, as essential data are missing regarding *Giardia* nuclei genomic organization, including ploidy. *Giardia* has one of the smallest eukaryotic genomes (11.7 Mb), which is divided into five chromosomes in a haploid set (Morrison et al. 2007). The chromosomes are variable in size, prone to subtelomeric rearrangements (Adam 1992; Hou et al. 1995; Prabhu et al. 2007) and extremely small but with typical chromatin condensation levels (Tumova et al. 2015). The ploidy of *Giardia* nuclei has remained unclear in past decades. The densitometry of the nuclear DNA content is considered to be valid even though an indirect method for ploidy evaluation revealed the tetraploidy of a *Giardia* cell (Bernander et al. 2001). In contrast, the imaging of chromosomes in individual nuclei indicated that the proposed diploid pattern per nucleus (2*n* = 10) can be modified to an aneuploid pattern and that the nuclei harbor different chromosome sets (Tumova et al. 2007). The structural organization of *Giardia* mitosis is unconventional and prone to chromatid missegregations (Tumova et al. 2015). The absence of key cell cycle regulators of a precise mitotic progression (Gourguechon et al. 2013; Vicente and Cande 2014) promotes conditions for aneuploidy development. Nevertheless, the extent and occurrence of aneuploidy in *Giardia* cell populations have not been satisfactorily documented.

In this study, we used a single‐cell approach to characterize karyotypes both within and between *Giardia* cell lines, that is, clinical isolates, different laboratory lines, and clones, all belonging to the same genetic group assemblage A. By direct chromosome counting and fluorescent *in situ* hybridization (FISH), we evaluated the ploidy and karyotype dynamics in different *Giardia* lines. The analyzed lines showed inter‐ and intraheterogeneous karyotype organization, different cell growth dynamics, and chromosomal missegregations. Thus, *Giardia* represents a novel noncancer model organism with an aneuploid genome that balances – within a single cell – the effects of different aneuploidy patterns occurring in the two nuclei.

## Experimental Procedures

### Cell lines and culturing conditions

Eight *Giardia intestinalis* lines from assemblage A were examined. Six of these lines originated from the same original WB isolate that was obtained in 1979 from a symptomatic metronidazole‐resistant patient by F.D. Gillin in Bethesda, USA (ATCC 30957), collected for this study from different sources. The line WB‐Meyer was a gift from Prof. E.A. Meyer (Oregon Health Sciences University, Portland, USA) in 1989, after which frozen in liquid nitrogen and then maintained in continuous culture since 2013; the line WB‐1W was a gift from Prof. C.C. Wang (University of California, San Francisco, USA) in 2001, after which frozen in liquid nitrogen and maintained in continuous culture since 2013; the line WB‐Tach was a gift from Prof. J. Tachezy (Charles University in Prague, Czech Republic) in 2013 and has been kept in continuous culture since then. The line WB‐ATCC was retrieved from the ATCC collection (www.lgcstandards-atcc.org) in 2012 and maintained in a continuous culture. Two laboratory lines of a WBc6 clone (ATCC 50803) of the original WB line were used: line WBc6‐Cande was a gift from Prof. Z.W. Cande (University of California, Berkeley, USA) in 2012 and kept in continuous culture, and line WBc6‐ATCC was retrieved from ATCC collection in 2013 and kept in continuous culture. The other clinical isolate, Portland‐1 (ATCC 30888), was isolated in 1971 by Prof. E.A. Meyer in Portland, USA, from a human patient; this line was retrieved from ATCC in 2012 and kept in continuous culture. The original Portland‐1 isolate was obtained in our laboratory in 1989 and, since then, has been maintained in continuous culture (labeled HP‐1). Axenic cultures were routinely maintained in TYI‐S‐33 medium (pH 6.8) in screw‐cap borosilicate glass tubes. The cultures were passaged twice per week by inoculating 500 *μ*L of the chilled 4‐day‐old culture into a new tube containing 7.5 mL of prewarmed TYI‐S‐33 medium. For all experiments, only the trophozoite life‐cycle stage of *Giardia intestinalis* was used unless otherwise stated.

### Cryopreservation, encystation/excystation, and cell growth measurement

For cryopreservation in liquid nitrogen, dimethyl sulfoxide was used as a cryoprotectant at a final concentration of 5% (v/v) in culture medium (Phillips et al. 1984). The in vitro encystation and excystation protocols were performed as previously described (Jirakova et al. 2012). The HP‐1 line was used for encystation/excystation experiments. To follow the growth dynamics of different *Giardia* lines, 2 mL of fresh culture with 1 × 10^4^ cells per milliliter was placed in separate wells of a 24‐well Nunclon© plate and maintained in quadruplicate at 37°C under anaerobic conditions (Oxoid AnaeroGen^™^, Oxoid Ltd.) for 24, 48, 72, 96, and 120 h. The cells were counted, and the growth curves were calculated.

### *Karyotype analysis of* Giardia intestinalis *lines*

Enrichment with mitotic cells was performed as previously described (Sagolla et al. 2006). For cytogenetic karyotyping, the chromosome suspensions were prepared, and the karyotypes were observed according to previously described protocols (Tumova et al. 2007, 2015). Briefly, hypotonization was achieved using 75 mmol/L KCl, and the time that was required for satisfactory chromosome separation varied from 15 to 45 min among *Giardia* lines, during which the cultures were kept at 37°C under anaerobic conditions. Then, the cells were centrifuged (870*g*, 6 min), and the pellet was fixed with a freshly prepared methanol/acetic acid fixative (3:1, v/v, repeated twice). The suspension was then dropped onto glass slides, air‐dried, mounted to DAPI/Vectashield and observed. The reliability of the counting method was ensured by random blind and independent counting by two researchers. The *Giardia* lines were examined several times, each from at least four different passages. The differences among the *Giardia* lines or among individual passages of a line were statistically tested using the Dell Statistica program (Pearson′s chi‐square test at a 0.05 significance level). The null hypothesis was that passages or lines have the same distribution of karyotype variants. To obtain relevant test results, the expected counts for each cross category (karyotype variant in each passage/line) must be greater than five in least 80% of the expected counts and greater than one in the remaining (Agresti 2002). Because we frequently observed minor karyotype variants with small occurrences, the condition of expected counts above five was not met; in such case, it is recommended to merge the categories (Agresti 2002). The term expected count regarding chi‐square testing refers to the expected frequencies in each cell of the contingence table if the null hypothesis is true. In this study (individually for each testing), we combined all karyotype variants in which at least one expected count for a passage/line was less than five. The chi‐squared test was performed on the combined data. To follow the lagging chromatids during *Giardia* mitosis, the chromatids were considered lagging if they failed to segregate poleward with the chromatid mass and were localized in the spindle midzone, separate from the daughter nuclei that formed at the poles (Thompson and Compton 2011; Tumova et al. 2015).

### Flow cytometry

Flow cytometry was performed as previously described (Uzlikova and Nohynkova 2014). Cell cultures of different *Giardia* lines were grown in separate 7.5 mL screw‐cap tubes and analyzed 1, 2, 3, 4, and 7 days after culture establishment. The cultures were set‐up as parallel screw‐cap tubes for each time interval to avoid abruption of the cell growth by a freezing phase used to detach the adhered trophozoites. An inoculum with the starting concentration 1 × 10^4^ cells per mL was taken from a stationary phase culture of the respective *Giardia* lines. The samples were measured using a BD FACSCanto^TM^ II instrument (BD). The events were recorded at a low speed of 200–400 events/s, and at least 10,000 events were recorded for each histogram. The data were analyzed using FACSDiva software.

### Fluorescent in situ hybridization

The FISH protocol was adapted from Conrad et al. (2011). Briefly, the chromosome spreads of the WBc6‐Cande line (10 + 10 chromosomes, passage px222, Table S2) were partially air‐dried, placed in 50% acetic acid solution for several seconds and then dried at 37°C. Prior to chromosome dehydration in a methanol series, RNase treatment (100 *μ*g/mL, Fermentas) to prevent unspecific probe‐RNA binding was applied for 60 min at 37°C, followed by three changes of 2× SSC for 5 min. The hybridization mixture contained 20 ng of labeled probe, 10 *μ*g of salmon sperm, and 50% deionized formamide (Sigma, St. Louis, MO) in 2× SSC and was applied at 82°C for 5 min. The single‐color FISH was developed by the TSA‐Plus TMR System according to the manufacturer′s directions (PerkinElmer, Waltham, MA) using a dig‐labeled probe and an anti‐dig‐HRP antibody (Roche Applied Science, Indianapolis, IN). In the two‐color FISH, a sequential double hybridization signal development was processed according to the manufacturer's directions (PerkinElmer, Waltham, MA), as a combination of (1) a dig‐labeled probe, anti‐dig‐HRP antibody, and TSA‐Plus TMR and (2) a biotin‐labeled probe, streptavidin‐HRP, and TSA‐Plus Fluorescein. An Olympus BX51 fluorescence microscope that was equipped with a DP70‐UCB camera was used for observation. For the two‐color FISH imaging, a Leica TCS SP2 AOBS confocal microscope was used. Single‐scan images were processed with Huygens Professional software using the classical maximum likelihood estimation deconvolution algorithm. For chromosome‐specific probes, we selected four single‐copy genes that were annotated in the *Giardia* database (http://giardiadb.org, line WBc6) to a chromosome four as follows: *rad50* GL50803_17495; *telomerase catalytic subunit (tert)* GL50803_16225; *isoleucyl‐tRNA synthetase* (*iso*) GL50803_104173; and *ubiquitin* (*ubi*) GL50803_13701. The probes *rad50* and *tert* were assigned to one chromosomal end (near 5′end), and the probes *ubi* and *iso* were assigned to the opposite chromosomal end (near 3′end). The probes against *actin* (*act*), chromosome 2, GL50803_40817, and a probe against *ser/thr phosphatase* (*ser*), chromosome 5, GL50803_7439, were used as controls for a specific probe binding to a chromosome of interest. The probe sizes, chromosome localization and primer sequences are listed in Table S5. The presence of a single copy of the respective probe sequence in the genome database was verified through reciprocal BLAST searches at http://giardiadb.org. The PCR products were cloned into the pJET 1.2/blunt cloning vector (Fermentas) and transformed into chemically competent TOP10 *E. coli* cells (Invitrogen). Purified PCR products that were amplified from plasmids that were isolated from a single bacterial colony (QIAprep Spin MiniprepKIT, Qiagen, Hilden, Germany) were labeled by random priming with digoxigenin‐11‐dUTP (Roche) or biotin‐11‐dUTP (PerkinElmer, Waltham, MA) using the DecaLabel DNA Labelling Kit (Fermentas). The probe binding efficiency was estimated at >300 cells as the percentage of the FISH‐labeled to unlabeled nuclei. This value varied among probes between 94% (*rad50*) to 98% (*ubi*) of the positive nuclei. Randomly chosen slides were counted by two persons, and the probe binding patterns were evaluated from at least three independent FISH experiments.

### Bioinformatic search for gene orthologs

The set of orthologs that were searched in the *Giardia* database (GiardiaDB) was designed as previously described (Aylon and Oren 2011) and included tumor suppressor p53 and its interactors: pRb, p21, p73, Lats2, BubR1, and p38. The searching procedure was previously described (Tumova et al. 2015). Briefly, *S. cerevisiae* and human protein sequences were used as queries for BLASTP in the GiardiaDB database. All of the found ORFs were employed for reciprocal BLASTP to evaluate the validity of the found results. The hits resulting from the initial search were further compared with all of the accessible protein sequences in the NCBI and Pfam databases to identify the domain organization. The presence/absence of these proteins in eukaryotes, or namely in *Giardia*, was previously evaluated (Rutkowski et al. 2010), (Manning et al. 2011). Therefore, the assessment of the presence of these proteins in *Giardia* was based on both our search and previously published findings.

## Results

### Karyotype heterogeneity in different Giardia lines (isolates, laboratory lines, and clones)

**Figure 1:**
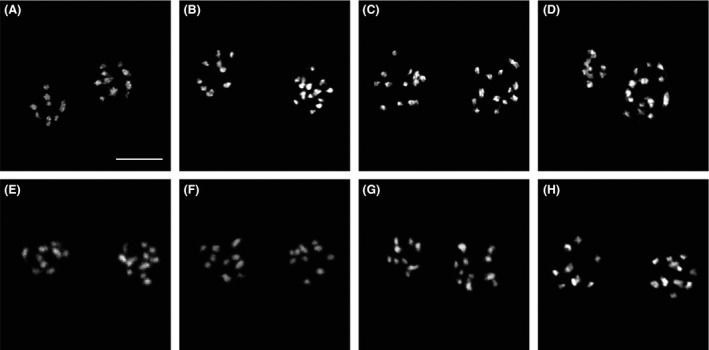
Condensed metaphase chromosomes in the two nuclei of different *Giardia intestinalis* lines. Metaphase chromosomes of representative cells from the respective *Giardia* lines (see Table 1) with the following prevailing karyotypes: (A) WB‐ATCC, 9 + 10 chromosomes; (B) WB‐1W, 10 + 14 chromosomes; (C) WB‐Tach, 13 + 13 chromosomes; (D) WB‐Meyer, 8 + 14 chromosomes; (E) WBc6‐ATTC, 9 + 11 chromosomes; (F) WBc6‐Cande, 10 + 10 chromosomes; (G) Portland‐1, 9 + 11 chromosomes; and (H) HP‐1, 9 + 11 chromosomes. The chromosomes were counterstained with DAPI. The frequency of the given karyotype pattern in the *Giardia* population and number of analyzed cells are listed in Table 1. Bar represents 5 *μ*m.

We compared six *Giardia* lines that were derived from the original WB isolate and two lines that were derived from the Portland‐1 isolate (Table 1 and Fig. 1). The expected chromosome number was 10 per nucleus, (i.e., karyotype 10 + 10), thus corresponding to the classic diploidy of a nucleus, having each of the five chromosomes in exactly two copies per nucleus. Because standard karyotyping does not permit the discrimination of individual chromosomes due to a lack of primary and secondary constrictions, the *Giardia* karyotype can be presented as two values corresponding to the chromosome numbers determined in each of the two nuclei (presented as x+y). The term “prevailing karyotype” refers to the most frequent karyotype variant for the given population and time. Moreover, minor karyotype variants were present in all of the examined *Giardia* lines. The “stable” *Giardia* lines did not show any change in the prevailing karyotype during long–term in vitro cultivation, in contrast to the “unstable” lines, where the prevailing karyotype changes were observed.

**Table 1:**
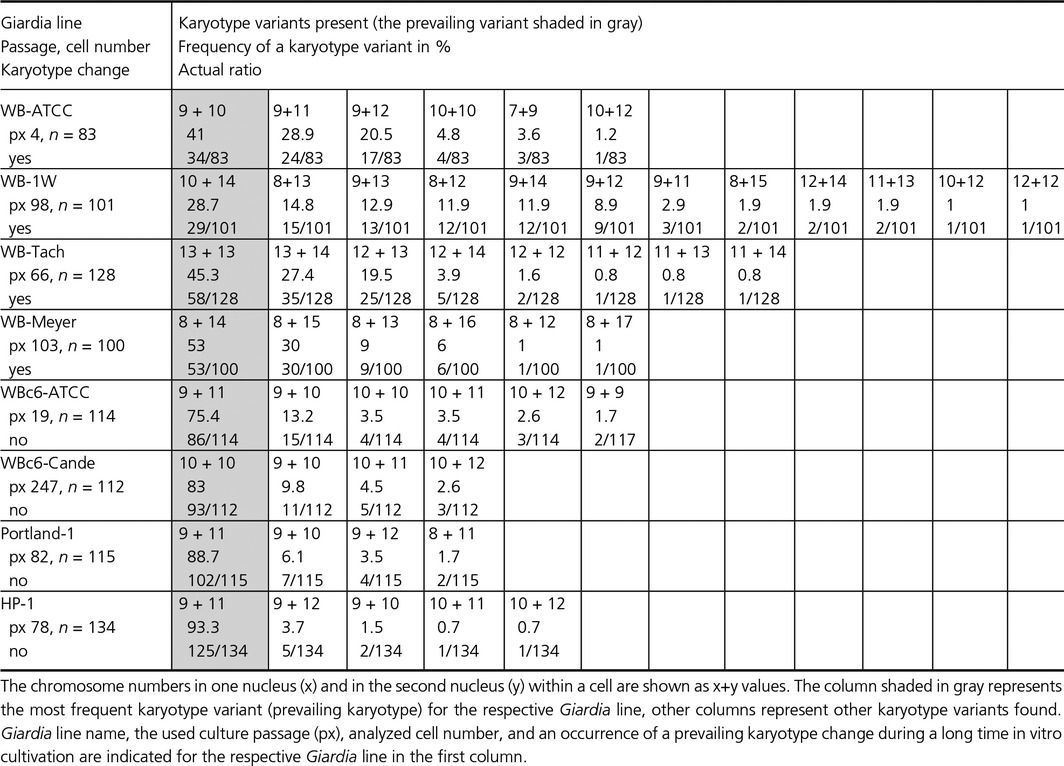
Chromosome numbers in different *Giardia intestinalis* lines (clinical isolates, laboratory lines, and clones)

Interestingly, gains/losses of chromosomes were observed from the expected 10 + 10 karyotype in the prevailing karyotypes of all of the *Giardia* lines, except for the WBc6‐Cande line. In the unstable lines, the karyotypes deviated more from the 10 + 10 pattern (WB‐1W 10 + 14, WB‐Tach 13 + 13, and WB‐Meyer 8 + 14) than in stable lines (WBc6‐Cande 10 + 10, WBc6‐ATCC 9 + 11, Portland‐1 9 + 11, and HP‐1 9 + 11). The tested lines significantly differed from each other in their karyotypes (Chi‐squared test with 28 df; the test statistics was equal to 1817 and near a 0 *P*‐value, and the test was performed on data from Table 1). The prevailing karyotypes of the studied lines differed in total chromosome number by up to 7 chromosomes, and the observed numbers ranged from 19 to 26 chromosomes per cell in the prevailing karyotype category, that is, the gains of chromosomes were more frequent than were chromosome losses. The two nuclei within a single cell differed by up to six chromosomes (Table 1, Fig. 1). The same chromosome numbers were found in the two nuclei only in the WBc6‐Cande (10 + 10) and WB‐Tach lines (13 + 13); in the latter, however, other prevailing karyotypes with different chromosome numbers in the two nuclei were observed in subsequent passages. Using karyotyping, we determined the *Giardia* karyotype as aneuploid‐near‐tetraploid with large karyotype heterogeneity among and within different *Giardia* lines.

### The genomic instability of Giardia karyotypes

**Figure 2:**
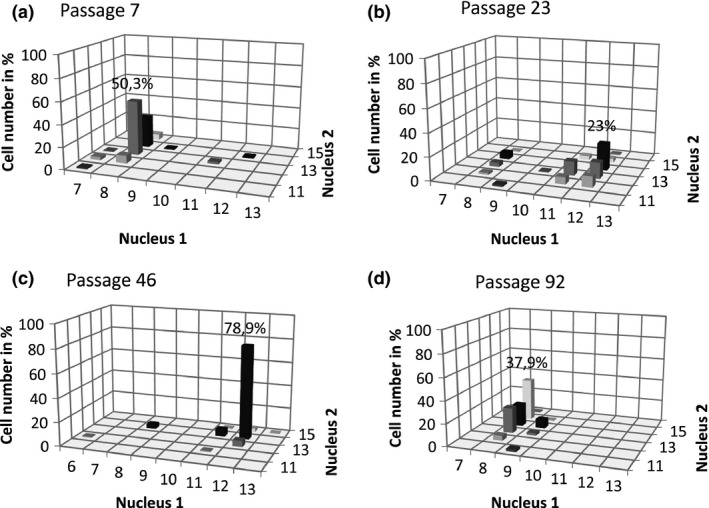
The karyotype changes in an unstable *Giardia intestinalis* line (WB‐Meyer) during a long‐term in vitro cultivation. (A‐D) Four selected passages in a one‐year observation period to show the dynamics in karyotype evolution and the karyotype change. The prevailing karyotype changed from 8 + 13 in passage 7 (A), to 12 + 14 in passage 23 (B, C), and 8 + 15 in passage 92 (D). Minor karyotype variants were present in all passages, reflecting the rapid karyotype evolution possibly due to missegregation. The frequency of some minor variants reached up to 30%. For the complete data, see Table S1. Representative images of karyotypes of cells with the prevailing karyotype patterns 8 + 13, 12 + 14, and 8 + 15 are shown in Fig. 3E‐G.

**Figure 3:**
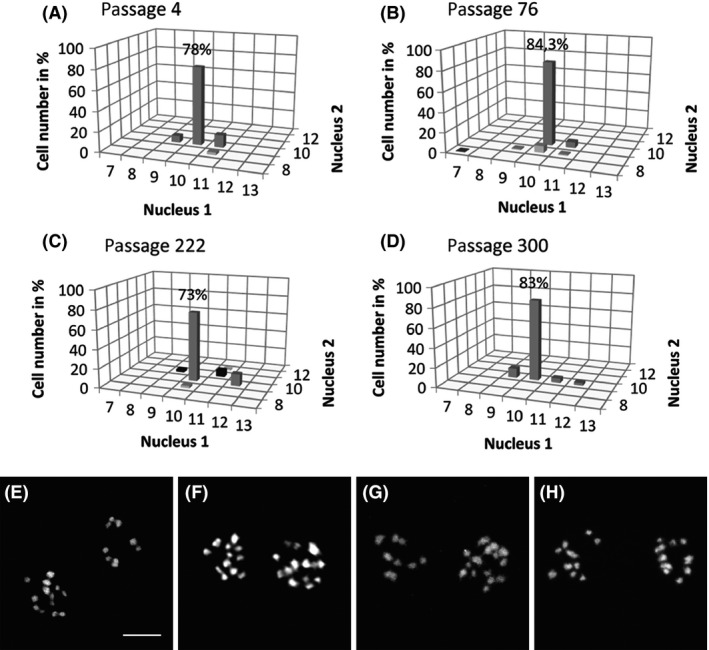
The karyotype stability in a stable *Giardia intestinalis* line (WBc6‐Cande) during a long‐term in vitro cultivation. (A‐D) Four selected passages in a two‐year observation period. The prevailing karyotype remained unchanged, that is, 10 + 10 chromosomes in all passages. Minor karyotype variants were generated, with some reaching up to a 13% frequency. For the complete data, see Table S2. (E‐G). Representative images of karyotypes of cells with the prevailing karyotype patterns from different WB‐Meyer passages (Fig. 2) as follows (E) px 7, 8 + 13, (F) px 23, 12 + 14, and (G) px 92, 8 + 15. (H) A representative image of a cell with the prevailing karyotype pattern in WBc6‐Cande with 10 + 10 chromosomes, observed in all analyzed passages. Bar represents 5 *μ*m.

The Portland‐1 isolate and its line HP‐1 revealed a stable aneuploid karyotype with a prevailing 9 + 11 pattern that has been maintained during 13 years of continuous laboratory cultivation. In contrast, the WB isolate and its laboratory lines, WB‐ATCC, WB‐1W, WB‐Tach, and WB‐Meyer, showed unstable karyotypes during in vitro cultivation (Fig. 2A‐D, Fig. 3E‐G), and all the analyzed passages of these lines statistically differed (Chi‐squared analysis of the dataset from Table S1, 24 df, test statistics 839, near 0 *P*‐value). Minor variants were newly generated, and some of them became subsequently prevalent. The karyotype of the WB‐Meyer line underwent a change from a prevailing karyotype 8 + 13 to 12 + 14, 8 + 15, and 8 + 14 within 16 months of observation. However, some WB lines with a clonal origin (WBc6‐ATCC and WBc6‐Cande) maintained stable karyotypes, and their prevailing karyotype did not change during long‐term cultivation (in WBc6‐Cande during 4 years of continuous cultivation) (Fig. 3A‐D, H). However, minor karyotype variants were generated in the WB clonal lines and the passages statistically differed from each other, indicating ongoing karyotype evolution even in the WB clonal lines (Chi‐squared analysis on data from Table S2, 6 df, test statistics 13.82, *P* = 0,0318). However, the differences in karyotype variant distributions among passages in a stable line WBc6‐Cande were smaller than in the unstable line WB‐Meyer, as apparent from the comparison of Figure 2 and Figure 3. Cryopreservation did not influence the karyotypes of stable lines (HP‐1 and WBc6‐Cande), and the in vitro encystation/excystation of the *Giardia* trophozoites of the line HP‐1 did not change its karyotype (9 + 11) in multiple experiments.

### Aneuploidy and unequal gene distribution detection in the two Giardia nuclei

To determine the chromosome copy number and to exclude the possibility that the observed odd chromosome numbers would refer to haploidy of the karyotype, we performed FISH experiments. For this, we selected WBc6‐Cande line, which revealed a stable karyotype with 10 + 10 chromosome numbers in 73% of cells and minor karyotype variants identified in 27% of cells (Table S2). For design of chromosome‐specific probes, we selected four single‐copy genes that were annotated in the *Giardia* database to a chromosome 4 as follows: *rad50* GL50803_17495; *telomerase catalytic subunit (tert)* GL50803_16225; *isoleucyl‐tRNA synthetase* (*iso*) GL50803_104173; and *ubiquitin* (*ubi*) GL50803_13701. The probes *rad50* and *tert* were assigned to one chromosomal end, and the probes *ubi* and *iso* were assigned to the opposite chromosomal end. Single‐copy gene probes were previously used for detecting *Giardia* chromosomes (Yu et al. 2002).

**Figure 4:**
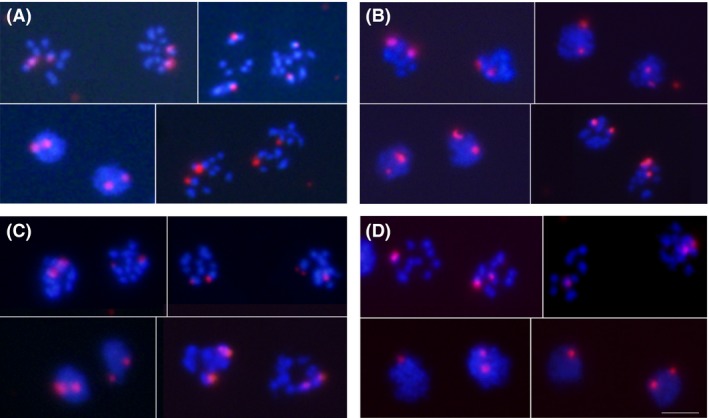
FISH analysis on chromosome 4 in *Giardia intestinalis* WBc6‐Cande line. Different FISH probes detecting chromosome 4 (red) hybridized on metaphase chromosomes and interphase nuclei. The DNA was counterstained with DAPI (blue). The 2 + 2 hybridization signals were detected in the majority of cells by using following FISH probes (A) *rad*, (B) *iso*, (C) *ubi*. (D) The t*ert* probe revealed the 2 + 1 binding pattern in the majority of cells, with the two signals localized in the more slowly condensing mitotic nucleus. The occurrences of probe binding patterns of the used probes are listed in Table S3. Bar represents 5 *μ*m. FISH, fluorescent *in situ* hybridization.

Using FISH hybridization on individual chromosomes and on interphase nuclei, the probes *rad50, iso,* and *ubi* detected 2 copies of chromosome 4 in each of the two nuclei in most of the cells (Fig. 4A‐C). On mitotic chromosome spreads, each signal labeled an individual chromosome; we can thus exclude that the doubled pattern would correspond to two chromatids of one chromosome in the interphase nucleus. The prevailing binding pattern in the two nuclei (2 +  2 signals) was detected in 60% of the cells using the *rad* probe (in 57.4% of cells using the *iso* probe and in 59% of the cells using the *ubi* probe); however, other patterns were also observed (2 + 1, 3 + 2, 1 + 1, 3 + 1) with approximately similar binding frequencies for the three probes (Table S3). Namely, for the *rad*/*iso*/*ub*i probes, respectively, there was a 2 + 1 pattern in 22.5/23.3/19.6% of the cells, a 3 + 2 pattern in 11/9.7/13.7% of the cells, 1 + 1 pattern in 6.5/3.2/1.9% of the cells and a 3 + 1 pattern in 0/6.4/5.8% of the cells. This indicates disomy of chromosome four in a *Giardia* nucleus in most of the cells; however, other copy numbers and/or chromosomal rearrangements leading to *rad50, iso,* and *ubi* gene duplications or losses were present in approx. 40% of the cells. The latter process was also indicated using the *tert* probe, which labeled the two nuclei with the 2 + 1 binding pattern in 45% of the cells, 2 + 2 (15%), 1 + 1 (14%), 3 + 2 (9%), 2 + 0 (8%), 3 + 1 (5%), and 3 + 0 (4%) (Fig. 4D). This result indicates a loss of a *tert* gene copy in one of the nuclei in the majority of the cells and indicates for the first time an unequal gene distribution between the two *Giardia* nuclei. Interestingly, in case of cells with the 2 + 1 *tert* probe binding pattern, one signal was always localized in the more rapidly condensing nucleus and the two signals in the slower nucleus (Fig. 4D). The slight asynchrony in chromosome condensation between the two nuclei was documented earlier; one of the two nuclei already had fully condensed chromosomes as defined particles, while in the other nucleus, the chromosome mass was still in the process of condensation (Tumova et al. 2007, 2015).

**Figure 5:**
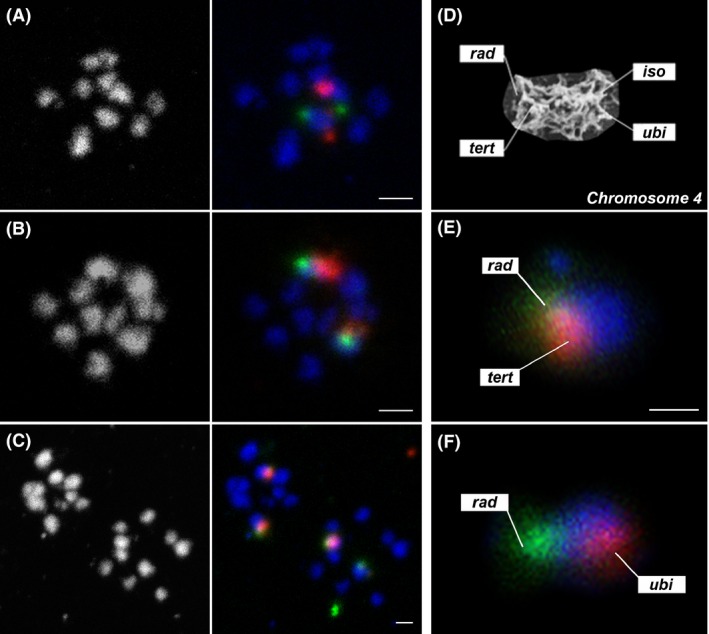
Two‐color FISH on chromosome 4 to reveal the probe specificity. Chromosome 4 was probed with two probes against opposite chromosome ends (A,B, F) and against the same chromosome ends (C, E). The probe localization is schematically shown in (D) on a chromosome 4 scanning‐electron‐micrograph (Tumova et al. 2015). Chromosomal spreads were counterstained with DAPI (blue), the red signal results from tetramethyl‐rhodamine‐TSA, the green signal results from fluorescein‐TSA. The used probes were (A) *iso* (red), *rad* (green), (B) *ubi* (red), *rad* (green), (C) *tert* (red), *rad* (green). The bar represents 2 *μ*m. Magnification of a chromosome with both probes hybridized to the same chromosome end (E) *tert* (red), *rad* (green), and to the opposite chromosome ends (F) *ubi* (red), *rad* (green). The bar represents 1 *μ*m. FISH hybridization of probes designed to different *Giardia* chromosomes can be found in Fig S1. FISH, fluorescent *in situ* hybridization.

Based on the 2 + 2 prevailing binding pattern observed on individual chromosomes 4, we can exclude a haploid chromosome set per *Giardia* nucleus. It means that the chromosome number different from 10 in most *Giardia* lines as shown by karyotyping, represents the whole‐chromosome aneuploidy. The diploidy of a nucleus (tetraploidy of a cell) is plausible in some, but not all, cells in the WBc6‐Cande line. The classic diploidy of a nucleus resulting in tetraploidy of a cell, however, must be verified by confirming the 2 + 2 pattern for all five of the *Giardia* chromosomes in the WBc6‐Cande line. Moreover, the loss of a *tert* gene copy in one of the nuclei reflects possible chromosomal rearrangements, which may lead to an unequal gene distribution between the two nuclei even in this *Giardia* line. The specificity of the used probes was also verified by two‐color FISH experiments, which permitted the specific binding of the probes to the opposite chromosome ends (Fig. 5A,B,F) or to the same chromosome ends (Fig. 5C and E) and to different chromosomes distinguished by their length (Fig. S1).

### *Comparison of cell proliferation characteristics in lines with stable and unstable karyotypes*

**Figure 6:**
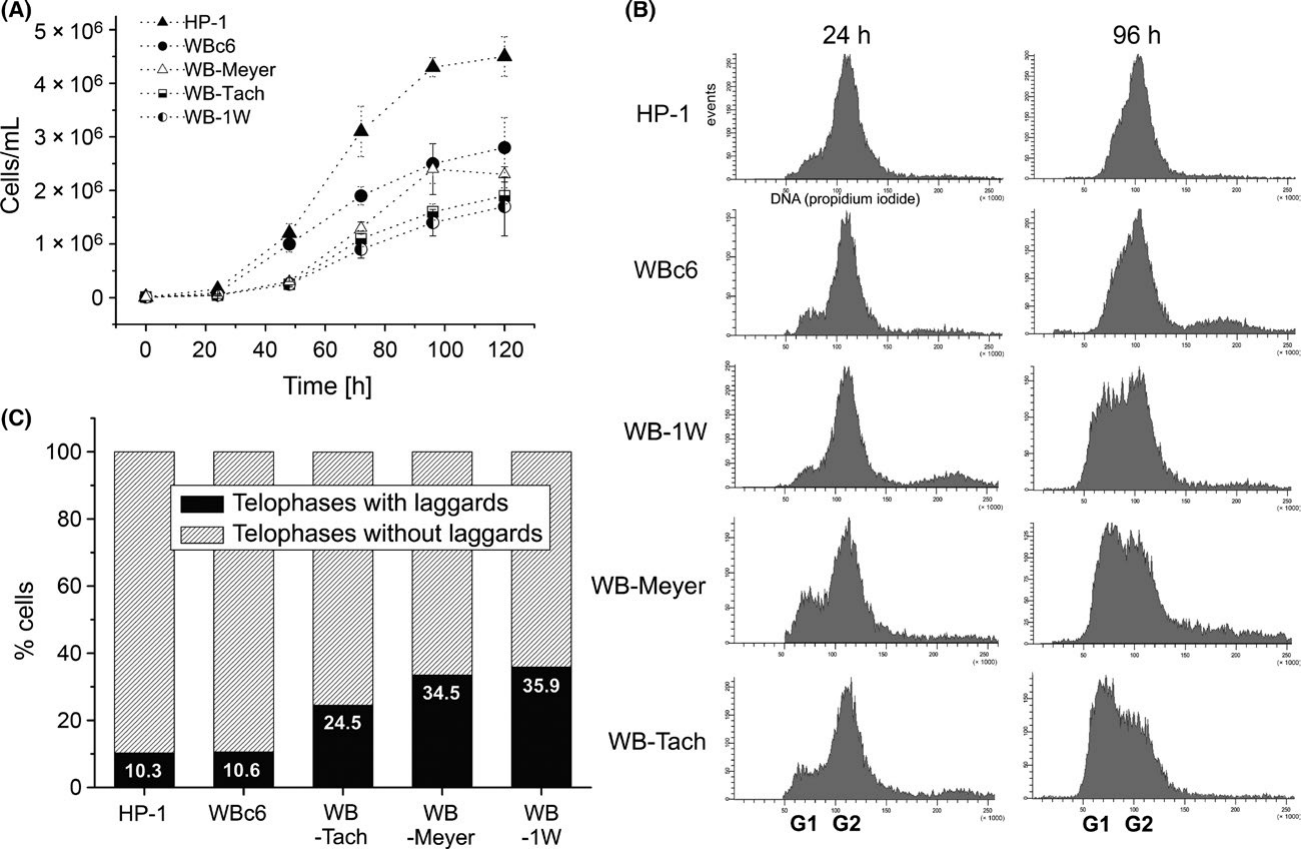
Phenotypic differences in *Giardia intestinalis* lines with stable and unstable aneuploid karyotypes. (A) The growth curves of *Giardia* lines revealed an earlier onset of a log‐phase in HP‐1 and WBc6‐Cande line compared to WB‐Meyer, WB‐Tach, and WB‐1W. The lines that derived from the original WB isolate reached a lower total cell count than did HP‐1, which derived from the Portland‐1 isolate. (B) DNA histograms from FACS analysis showing the proliferative phases in different *Giardia* lines. The HP‐1 and WBc6‐Cande stable lines underwent the proliferative phase characterized by approximately the same cell number in G1 and G2 after 48 and 72 hr (see Fig S2), respectively, and reached after 96 hr the stationary phase characterized by solely the G2 peak. In contrast, the unstable aneuploid lines (WB‐1W, WB‐Meyer, WB‐Tach) revealed the active proliferation in later phases of in vitro cultivation (after 96 h). For all time intervals, see Fig S2. (C) The frequency of lagging chromatids between the formed daughter telophase nuclei observed in cytogenetic preparations.

Aneuploidy is usually detrimental in eukaryotic cells. A proliferative disadvantage was evident in *Giardia* lines with unstable and highly aneuploid karyotypes compared to lines with the stable karyotype, as judged by the growth curve (Fig. 6A) and FACS analysis (Fig. 6B). The stable lines HP‐1 and WBc6‐Cande revealed an earlier onset of the logarithmic phase of growth, although the two lines belonging to different clinical isolates differed in the final total cell count. In contrast, the unstable WB lines (WB‐1W, WB‐Meyer, WB‐Tach) revealed a decreased total cell count and a later onset of the proliferative log‐phase. As clear from the FACS histograms, the unstable lines revealed an enrichment of cells in the G1 and S phases in later intervals (even after 96 h of culture establishment) due to the presence of cells actively progressing through the cell cycle (Fig. 6B). Furthermore, the stationary phase and G2 arrest were reached earlier in the lines with a stable aneuploid karyotype than in the unstable aneuploid lines (Fig. 6B, Fig. S2). Thus, an unstable karyotype in *Giardia* hampers cell proliferation.

### *Chromosome missegregations as the cause of whole‐chromosome aneuploidy in* Giardia

**Figure 7:**
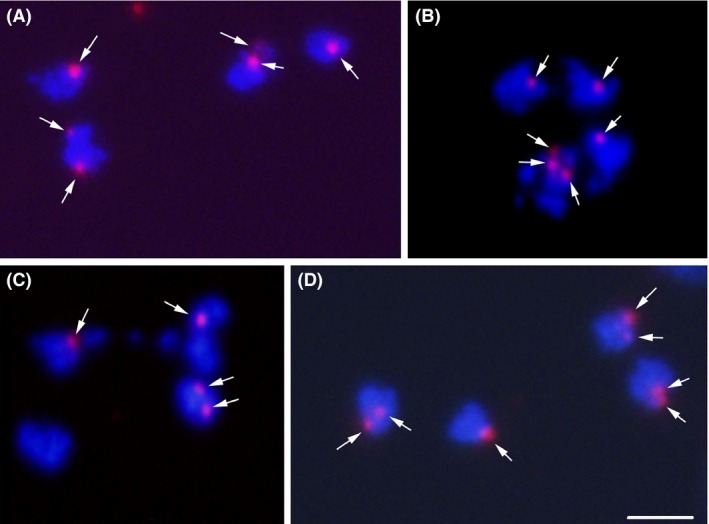
Chromatid missegregations detected by FISH on daughter nuclei. The *tert* probe was hybridized on quartets of daughter nuclei. The two mother nuclei migrated one above the other and segregated laterally, as described previously (Tumova et al. 2007). The chromatin was counterstained with DAPI (blue). The *tert* probe binding pattern (red) is shown by arrows. We observed symmetric chromatid segregation to daughter cells generated from a mother cell with putatively 2 + 1 initial pattern, that is, 2 + 1 and 2 + 1 pattern (A), as well as asymmetric patterns, daughter cells with 3 + 1 and 1 + 1 patterns (B), 2 + 1 and 0 + 1 patterns from a mother cell with a putative 1 + 1 initial pattern (C). Uneven number of chromatids resulted in their uneven distribution (2 + 1 and 2 + 2) (D). Bar represents 5 *μ*m. FISH, fluorescent *in situ* hybridization.

The *Giardia* lines differed in the frequency of lagging chromatids, which generally correlates with missegregations and chromosomal instability. The lines with the unstable karyotype revealed an approx. threefold increase in the number of laggards in comparison with lines with the stable karyotype. More than 30% of the laggards were observed in WB‐Tach, WB‐1W, and WB‐Meyer, whereas fewer than 11% of the laggards were observed in WBc6‐Cande and HP‐1 (Fig. 6C). To gain insight into the mechanisms that might generate the observed aneuploidy, we used FISH to detect the chromatid segregation to daughter nuclei during mitosis. We observed three segregation patterns: (1) even numbers of chromatids segregating evenly to daughter nuclei (Fig. 7A), (2) even numbers of chromatids segregating unevenly to daughter nuclei (Fig. 7B and C), and (3) uneven numbers of chromatids segregating unevenly to daughter nuclei (Fig. 7D).

### Tolerance to aneuploidy

To understand the mechanisms underlying further proliferation of the aneuploid *Giardia* cells, we aimed to analyze genes that were previously shown to be involved in the response to aneuploidy. The genome of *Giardia* does not contain genes for the key tumor suppressor homologs p53 and p73 (p53 family member), which are usually responsible for the slow proliferation and G1 arrest of aneuploid cells. This result is consistent with findings that these regulators evolved first in Choanozoans from the Opisthokonta group (Rutkowski et al. 2010). p21 and pRb are also absent in *Giardia*. The search and bioinformatic study on the *Giardia* kinome published by Manning et al. (2011) confirm the absence of Mad3/BubR1, a spindle checkpoint protein and a p53‐interacting partner. The same study describes the presence of a member of the AGC NDR kinase family (GL 50803_8587) that corresponds to the best candidate identified in this study when searching for a Lats2 ortholog. When searching for a p38 ortholog (syn. MAPK14), a member of the CMGC MAPK kinase family was found (GL50803_17563). The search results are summarized in Table S4. Both Lats2 and p38 act upstream of p53 in eukaryotes, where they trigger apoptosis and induce stress pathways. However, the role of the identified kinases in *Giardia* ploidy control remains unclear. Taken together, *Giardia* lacks most of the factors that are involved in the response to aneuploidy in higher eukaryotes.

## Discussion

*Giardia intestinalis* is an important human and veterinary pathogen causing diverse arrays of manifestations from asymptomatic infections to chronic malabsorptive diarrhea. Different disease outcomes are, together with variable host susceptibility, typically linked to the genetic diversity of the pathogen species (Shah et al. 2005). However, the sources of genetic diversity in *Giardia* remain unknown.

Genetic diversity among *Giardia* clinical isolates has been studied by many groups following the identification of different haplotypes through multilocus sequencing, protein polymorphisms (Monis et al. 2003) and PFGE profiles (Le Blancq et al. 1991). Additionally, the difference in allelic sequence heterogeneity was revealed from sequencing projects, including greater than a log difference between the closely related isolates (Adam et al. 2013) and a 200‐fold difference in heterozygosity between the lowest and highest values (Franzen et al. 2009; Jerlstrom‐Hultqvist et al. 2010). The sequence heterogeneity in the three genotyped loci together with enzyme electrophoretic studies led to the establishment of eight genetic groups called assemblages (A‐H), which display to some extend a different host specificity (Mayrhofer et al. 1995; Monis et al. 1999). A very low frequency of recombination between assemblages led to the suggestion of their genetic isolation and mutual speciation (Xu et al. 2012). Nevertheless, a correlation between assemblage and disease, drug susceptibility or proliferation has yielded conflicting results (Monis et al. 2009; Benere et al. 2011). Moreover, the proteomic studies of virulent and avirulent clinical isolates belonging all to assemblage A indicate the presence of an underlying intra‐assemblage genetic diversity of *Giardia* isolates, independent of the assemblage‐specific sequence heterogeneity (Emery et al. 2014, 2015).

In this study, we suggested a new mechanism for evolving genetic diversity with a focus on intra‐assemblage and intra‐isolate genetic variation in *Giardia*. We reveal aneuploidy as a widespread feature of this single‐celled eukaryote. We first described the aneuploidy and genetic diversity of the two nuclei in four *Giardia* lines (Tumova et al. 2007). In this study, more profound chromosome number changes were identified in additional *Giardia* lines, differing by up to seven chromosomes between lines. In the prevailing karyotype category, the two nuclei within one cell differed up to six chromosomes. An altered copy number of *Giardia* chromosomes was previously referred to from physical mapping by PFGE as a duplication of the 1.78 Mb chromosome (Chen et al. 1994). Aneuploidy in *Giardia* remained hidden for a long time for the following reasons. First, PFGE, as the most frequently used method for karyotype analysis in *Giardia*, detects chromosome size variation rather than a chromosome copy number. Second, the *Giardia* genome has so far been studied by using bulk populations, thus masking heterogeneity and reflecting only the total chromosome or gene dosage in the population. This approach was applied for gene/contig assembly to chromosomes, gene copy number evaluation, analysis of SNPs, and transcriptomics (Adam 1992; Morrison et al. 2007; Birkeland et al. 2010; Faghiri and Widmer 2011; Tolba et al. 2013). In *Giardia*, next‐generation sequencing could be a sensitive tool to detect aneuploidy as copy number variation calculated from the depth of mapped reads (Wang et al. 2014), as has been documented in *Leishmania* populations, another parasitic protist with a constitutive aneuploidy (Downing et al. 2011; Rogers et al. 2011). We applied a single‐cell‐oriented approach by evaluating the karyotypes of individual cells and individual nuclei within one cell. In all *Giardia* lines, even in populations derived from biological clones, a considerable karyotype heterogeneity was observed as evidenced by the presence of minor karyotype variants. The frequency of these minor variants changed during long‐term observation, and in the cell lines with unstable aneuploidy, the minor variants outgrew the dominant karyotype and became the prevailing karyotype of the population. Evolving chromosome size variation during a short period of in vitro passaging of the *Giardia* WB isolate has already been observed (Le Blancq et al. 1992) and accounted for mutations in rRNA‐encoding areas and other loci. Thus, the karyotype dynamics comprising both the numerical and structural variations is exceptional and has uncovered the so‐far‐undetected potential for genetic diversity evolution in *Giardia*. Similar features of chromosomal instability and population heterogeneity were observed, for example, in cancer cells by creating and propagating minor genetic variants that can outgrow the majority under specific conditions (Maley et al. 2006; Sipos et al. 2014).

We suggest that these karyotype variants in *Giardia* may arise by mitotic errors due to defective chromosome segregation. Missegregations (chromosome nondisjunctions) due to the uncorrected merotelic kinetochore‐microtubule attachments are considered the major mechanistic cause of aneuploidy in cancer cells (Thompson and Compton 2011). Additionally, there is no SAC checkpoint control resulting in mitotic arrest to correct aberrant or lacking microtubule‐kinetochore attachment in *Giardia* (Vicente and Cande 2014). Reduced error correction and altered centromere cohesion leads to anaphase laggards, as found in trypanosomes (Gluenz et al. 2008) and higher eukaryotes (Diaz‐Martinez et al. 2010; Lane and Clarke 2012). In *Giardia*, an alternative cohesin complex composition lacking the Rad21/Scc1 ortholog, the absence of securin, Sgo1, and all APC components for regulated sister chromatid separation (Gourguechon et al. 2013; Tumova et al. 2015) may further contribute to missegregation and aneuploidy. The organization of *Giardia* anaphase is atypical due to a spatial chromatid segregation in rows between spindle poles (Tumova et al. 2015). Because mitosis in *Giardia* occurs in a closed nuclear membrane (Sagolla et al. 2006; Tumova et al. 2007), a missegregation event does not lead to micronuclei formation. Instead, the lagging chromatid remains inside the nuclear membrane and might be pulled to the other daughter nucleus. In a eukaryotic cell, the merotelic spindle attachment is usually conditioned by an excess of microtubules (Lane and Clarke 2012). However, this is not the case for *Giardia*, where a single intranuclear microtubule likely attaches to a single chromatid (Dawson et al. 2007; Tumova et al. 2007). The precise spindle microtubule‐kinetochore interface in *Giardia* is not known. In *Giardia*, the depletion of Mad2, Bub3, and Mps1 generates defects in chromatid segregation after a morpholino‐mediated gene knockdown (Vicente and Cande 2014). Whether the observed differences between *Giardia* lines in laggard frequency are conditioned by different gene expression, the gain or loss of mitotic regulators remains enigmatic. Thus, more effort is required to understand the mechanistic cause of the incorrect chromatid segregation in *Giardia*. The mechanisms underlying aneuploidy development in lower eukaryotes can be quite unexpected. Interestingly, the RNAi knockout of nucleoporin TbMlp2 disrupts the mitotic distribution of chromosomes in *Trypanosoma brucei*, leading to a well‐tolerated aneuploidy (Morelle et al. 2015). In *Leishmania*, a process called asymmetric chromosomal allotment was hypothesized from the proportions of mono‐, di‐, and trisomic cells (Sterkers et al. 2011) and was explained by a model of defective chromosomal replication, leading to the over‐ and underreplication of chromatids and to supernumerary or missing chromosomes, respectively, in the next cell generation (Sterkers et al. 2012). Whether this replication defect is also present in *Giardia* and leads to aneuploidy development remains to be elucidated. The different segregation patterns that have been observed in *Giardia* allow for the consideration of both segregation and replication defects.

Regarding the tolerance of aneuploidy, the bioinformatic analysis that was conducted in this study revealed that further aneuploid cell proliferation in *Giardia* can be facilitated by the lack of multiple checkpoint factors. Tolerance to aneuploidy in higher eukaryotes is usually mediated by the loss or inactivation of tumor suppressors (Andreassen et al. 2001; Margolis et al. 2003). The key tumor suppressor molecules are not encoded in the *Giardia* genome (p53, p73, pRb, and p21), and pathways to inhibit the proliferation of aneuploids by a G1 cell cycle arrest have not been identified and may be absent from its genome. As a consequence, aneuploidy in *Giardia* is further propagated and represents a constitutive feature of its karyotype.

It remains unclear what adaptation strategies are necessary to support the proliferation of cells with aneuploidy and the imbalanced genomic content, as aneuploidy usually presents detrimental conditions for cell physiology (Gordon et al. 2012). The reduction in fitness of aneuploid cells usually comprises growth deficiencies, G1 delay, elevated sensitivity to stress conditions, protein stoichiometry imbalance, and impaired protein folding in yeast and mammalian cultures (Torres et al. 2007; Williams et al. 2008; Donnelly and Storchova 2014). Concomitantly, advantages associated with the gain or loss of chromosomes, such as drug resistance acquisition and growth‐promoting genetic alterations, have been shown in yeast and *Candida* sp. (Selmecki et al. 2006; Pavelka et al. 2010). The presence of originally four chromosome sets in *Giardia* cell can be on one hand a natural prerequisite enabling aneuploidy evolution – the tetraploid cells are generally considered an intermediate stage on route to aneuploidy and cancer (Storchova and Pellman 2004). It should be noted that in case of *Giardia,* two originally diploid nuclei occur within one cell instead of one tetraploid nucleus. On the other side, as a result of the presence of multiple chromosome sets, which might balance the altered chromosome and gene dosage, small‐scale chromosome copy number changes may not influence *Giardia* physiology to a large extent. The detrimental effects of aneuploidy have been shown to have a greater impact on haploid yeast cells (Torres et al. 2007) compared to diploid or polyploid yeast lines (Storchova et al. 2006). In our first efforts to evaluate aneuploidy impact on a *Giardia* cell, we compared *Giardia* lines with aneuploid stable and unstable karyotypes. The unstable lines had increased karyotype heterogeneity, novel karyotype variants occurrence, lagging chromatids production, and modified kinetics of cell cycle progression and proliferation. The observed chromosomal instability in these *Giardia* lines seems to have had rather detrimental effects that cannot be easily balanced compared to *Giardia* lines with stable aneuploidy and few missing/supernumerary chromosomes. Interestingly in the stable *Giardia* lines, a so far unrecognized biological mechanism impedes an excessive drift of aneuploidy and ensures stable transmission of a respective aneuploid karyotype pattern to next generations for many years of in vitro cultivation.

It is difficult to interpret the impact of aneuploidy in *Giardia* on the cell fitness regarding the host–parasite interaction as either detrimental or beneficial. While the rushed proliferation in the stable aneuploid lines may be beneficial for rapid intestinal microvilli colonization, the increased chromosome missegregation leading to new karyotype variants in the unstable lines may confer selective advantages during interactions with the host immune system. Notably, this variable pathogenic potential was observed in the *Giardia* cell lines (laboratory lines and clones) that were derived from one original clinical drug‐resistant isolate WB. This finding raises questions regarding the development of efficient therapeutic strategies. The karyotype heterogeneity and evolution during in vitro cultivation also set up additional requirements for researchers working with this pathogen. The observed genomic instability as well as the tendency to generate new karyotype variants and to outcompete the other karyotypes can all hinder the gene knock‐out and gene‐editing efforts in *Giardia*, which has so far been unsuccessful in this eukaryotic model.

## Conflict of Interest

The authors declare no potential conflicts of interest.

## Supporting information


**Figure S1**. FISH hybridization of probes designed to different *Giardia intestinalis* chromosomes.

**Figure S2**. FACS analysis of cell cycle progression in *Giardia intestinalis* lines with stable and unstable aneuploid karyotypes.

**Figure S3**. Proposed model for the karyotype evolution toward aneuploidy in *Giardia intestinalis*.

**Table S1**. Observed karyotype variants of the WB‐Meyer line during a long‐term in vitro cultivation.

**Table S2**. Observed karyotypes of the WBc6‐Cande line during a long‐term in vitro cultivation.

**Table S3**. The table shows the percentages of probe binding patterns in the two *Giardia* nuclei observed by single‐color FISH in interphase and mitotic nuclei. *N* indicates the number of cells, in which the binding pattern was evaluated.

**Table S4**. Outcomes of a bioinformatic search for orthologs of major tumor suppressor factors and checkpoint activators implied in the ploidy control.

**Table S5**. Information regarding the probes used in FISH experiments on *Giardia intestinalis* chromosomes.
