# Supplementary figures and images for: Constitutive aneuploidy and genomic instability in the single‐celled eukaryote Giardia intestinalis
Source: Microbiologyopen. 2016 Mar 23;5(4):560–74. doi: 10.1002/mbo3.351 (PMC4985590; doi:10.1002/mbo3.351)

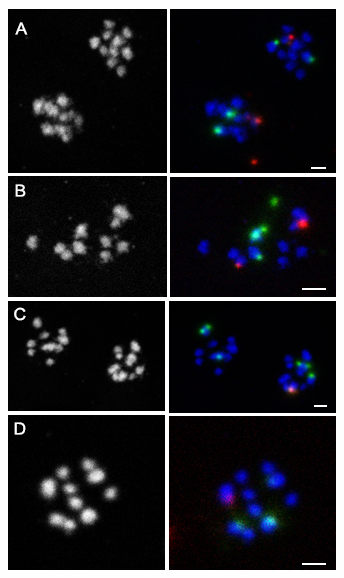

Supplement: Supplementary file 1 — Figure S1. FISH hybridization of probes designed to different Giardia intestinalis chromosomes. [file MBO3-5-560-s001.tif]

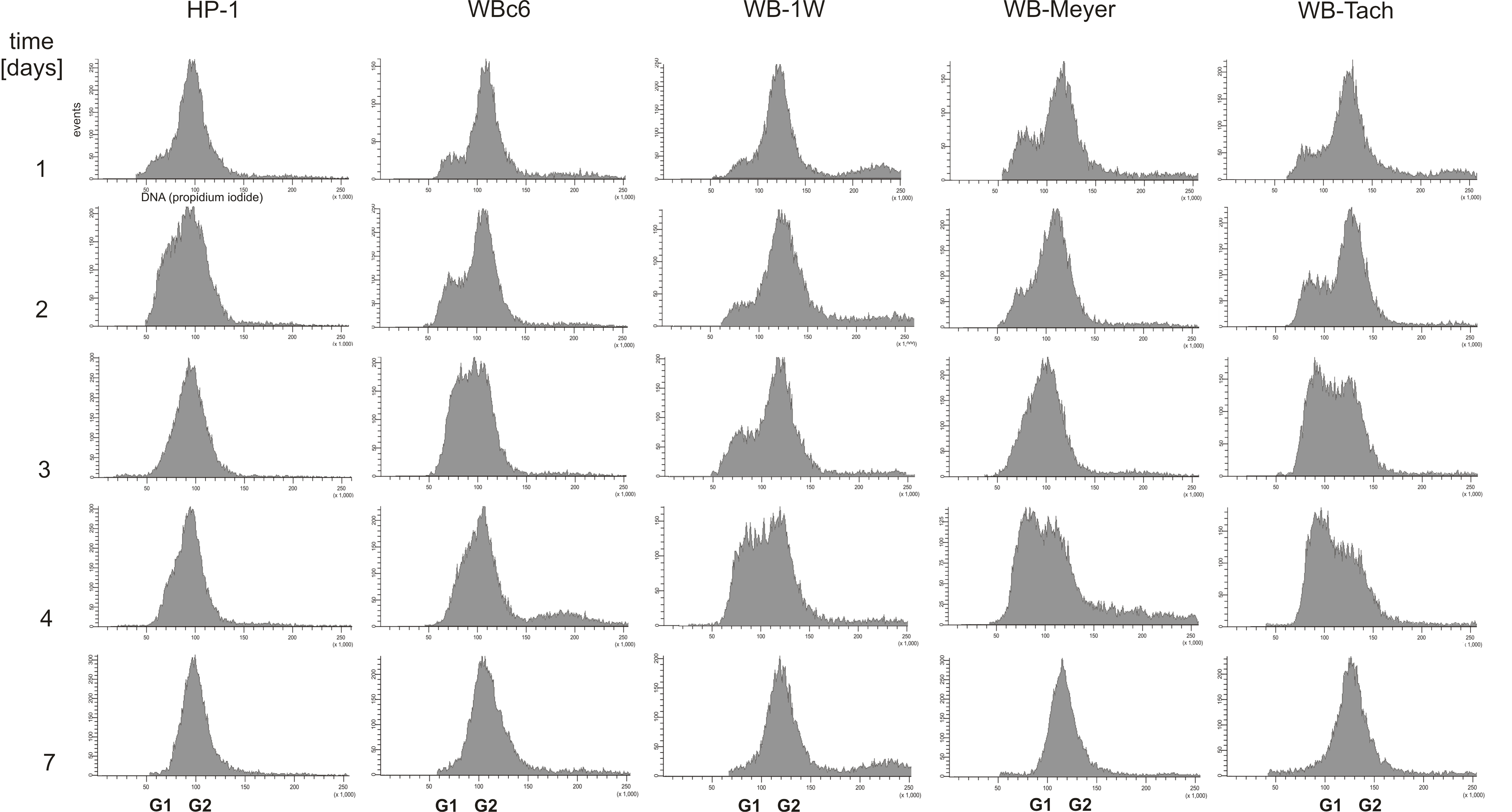

Supplement: Supplementary file 2 — Figure S2. FACS analysis of cell cycle progression in Giardia intestinalis lines with stable and unstable aneuploid karyotypes. [file MBO3-5-560-s002.JPG]

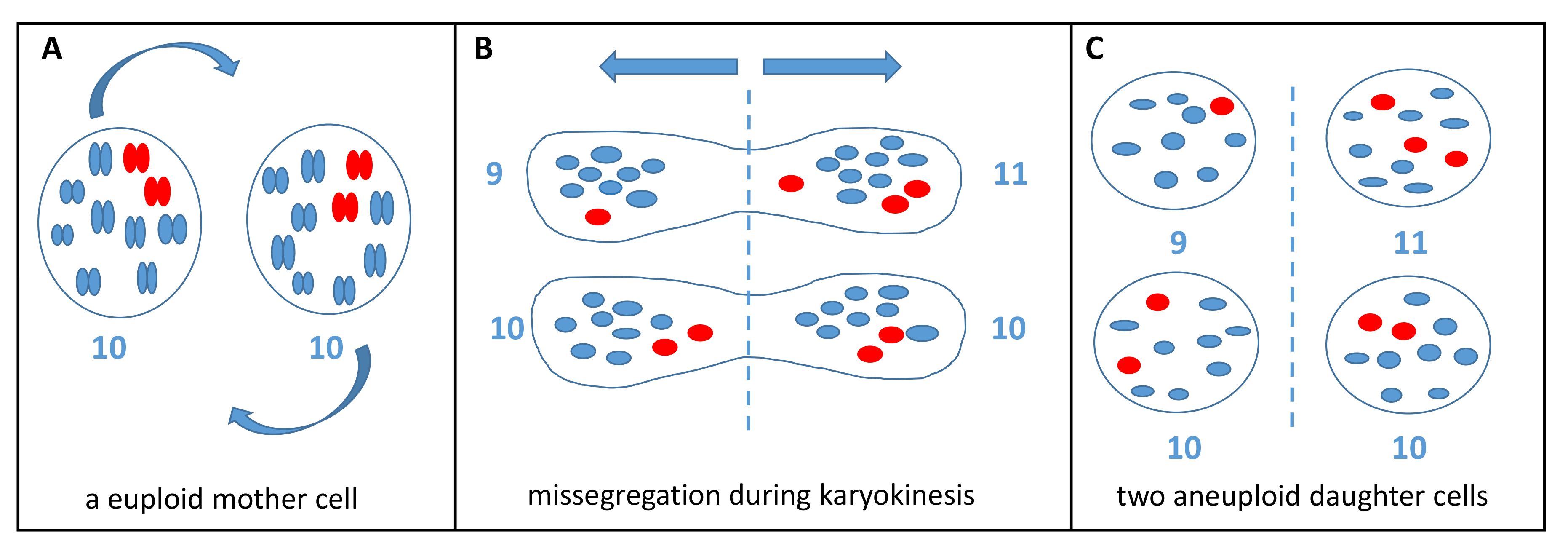

Supplement: Supplementary file 3 — Figure S3. Proposed model for the karyotype evolution toward aneuploidy in Giardia intestinalis. [file MBO3-5-560-s003.tif]
