# Supplementary material for: Constitutive aneuploidy and genomic instability in the single‐celled eukaryote Giardia intestinalis
Source: Microbiologyopen. 2016 Mar 23;5(4):560–74. doi: 10.1002/mbo3.351 (PMC4985590; doi:10.1002/mbo3.351)
Supplement: Supplementary file 4 — Table S1. Observed karyotype variants of the WB‐Meyer line during a long‐term in vitro cultivation. [file MBO3-5-560-s004.docx]

Table S1

Observed karyotype variants of the WB-Meyer line during a long-term *in vitro* cultivation.

| Passage  (date) | Karyotype | | | | | | | | | | | | | | | | | |
| --- | --- | --- | --- | --- | --- | --- | --- | --- | --- | --- | --- | --- | --- | --- | --- | --- | --- | --- |
|  | Frequency in % | | | | | | | | | | | | | | | | | |
|  | Number/total number | | | | | | | | | | | | | | | | | |
| px 7  (14.03.13) | **8+13**  50,3  85/169 | **8+14**  28,9  49/169 | **8+12**  7,1  12/169 | **8+15**  5,3  9/169 | **7+12**  2,9  5/169 | **11+13**  1,8  3/169 | **7+11**  1,2  2/169 | **12+14**  1,2  2/169 | **7+13**  0,6  1/169 | **9+14**  0,6  1/169 |  |  |  |  |  |  |  |  |
| px 19  (10.05.13) | **12+14**  26,8  48/179 | **8+14**  17,8  32/179 | **8+12**  11,8  21/179 | **8+13**  10,6  19/179 | **8+15**  7,3  13/179 | **12+13**  6,1  11/179 | **11+13**  5,6  10/179 | **9+14**  2,8  5/179 | **12+12**  2,2  4/179 | **12+15**  2,2  4/179 | **10+14**  1,7  3/179 | **7+13**  1,7  3/179 | **9+12**  1,1  2/179 | **13+14**  0,6  1/179 | **10+13**  0,6  1/179 | **11+15**  0,6  1/179 | **9+13**  0,6  1/179 |  |
| px 23  (28.05.13) | **12+14**  24  28/117 | **12+13**  13,7  16/117 | **11+13**  12  14/117 | **11+14**  10,3  12/117 | **12+12**  9,4  11/117 | **8+14**  6  7/117 | **11+12**  6  7/117 | **8+13**  3,4  4/117 | **12+15**  3,4  4/117 | **11+15**  1,7  2/117 | **9+11**  1,7  2/117 | **8+12**  1,7  2/117 | **9+12**  1,7  2/117 | **10+12**  1,7  2/117 | **12+16**  0,9  1/117 | **8+15**  0,9  1/117 | **10+13**  0,9  1/117 | **11+18**  0,9  1/117 |
| px 32  (16.07.13) | **12+14**  53,3  73/137 | **11+14**  15,3  21/137 | **8+14**  10,2  14/137 | **12+13**  8  11/131 | **8+13**  4,4  6/137 | **12+15**  3  4/137 | **10+15**  2,2  3/137 | **8+11**  2,2  3/137 | **11+15**  1,5  2/137 |  |  |  |  |  |  |  |  |  |
| px 46  (20.09.13) | **12+14**  78,9  105/133 | **11+14**  6  8/133 | **12+13**  5,2  7/133 | **8+14**  3,7  5/133 | **12+15**  3  4/133 | **11+15**  0,8  1/133 | **11+12**  0,8  1/133 | **13+15**  0,8  1/133 | **6+12**  0,8  1/133 |  |  |  |  |  |  |  |  |  |
| px 92  (9.5.14) | **8+15**  37,9  52/137 | **8+13**  23  32/137 | **8+14**  20,4  28/137 | **9+14**  8  11/137 | **8+12**  4,3  6/137 | **9+11**  2,2  3/137 | **9+13**  2,2  3/137 | **9+15**  0,7  1/137 | **8+16**  0,7  1/137 |  |  |  |  |  |  |  |  |  |
| Px103  (10.7.14) | **8+14**  53  53/100 | **8+15**  30  30/100 | **8+13**  9  9/100 | **8+16**  6  6/100 | **8+12**  1  1/100 | **8+17**  1  1/100 |  |  |  |  |  |  |  |  |  |  |  |  |

The chromosome numbers in one nucleus (x) and in the second nucleus (y) within one cell are shown as x+y values. The column shaded in grey represents the most frequent karyotype variant (prevailing karyotype) for the respective WB-Meyer passage. Other columns represent other karyotype variants found. The passage number (px) and the date of analysis are indicated in the first column for the WB-Meyer passage. The prevailing karyotype change occured from passage 7 to passage 19 (8+13 to 12+14), from passage 46 to 92 (12+14 to 8+15) and from passage 92 to 103 (8+15 to 8+14) during the one-year-observation period.
