# Supplementary material for: Constitutive aneuploidy and genomic instability in the single‐celled eukaryote Giardia intestinalis
Source: Microbiologyopen. 2016 Mar 23;5(4):560–74. doi: 10.1002/mbo3.351 (PMC4985590; doi:10.1002/mbo3.351)
Supplement: Supplementary file 5 — Table S2. Observed karyotypes of the WBc6‐Cande line during a long‐term in vitro cultivation. [file MBO3-5-560-s005.docx]

Table S2

Observed karyotypes of the WBc6-Cande line during a long-term *in vitro* cultivation.

| Passage  (date) | Karyotype | | | | | | | | |
| --- | --- | --- | --- | --- | --- | --- | --- | --- | --- |
|  | Frequency in % | | | | | | | | |
|  | Number/total number | | | | | | | | |
| px 4  (1.2.12) | **10+10**  78  60/77 | **10+11**  12,3  10/77 | **9+10**  6,5  5/77 | **9+11**  2,6  2/77 |  |  |  |  |  |
| px 76  (16.10.12) | **10+10**  84,3  145/172 | **9+10**  6,9  12/172 | **10+11**  5,8  10/172 | **9+11**  1,1  2/172 | **9+9**  1,1  2/172 | **7+9**  0,6  1/172 |  |  |  |
| px 222  (23.10.13) | **10+10**  73  122/166 | **10+12**  12,6  21/166 | **10+11**  7,2  12/166 | **9+11**  2,9  5/166 | **9+10**  2,4  4/166 | **11+12**  1,2  2/166 |  |  |  |
| px 300  (10.07.14) | **10+10**  83  93/112 | **9+10**  9,8  11/112 | **10+11**  4,5  5/112 | **10+12**  2,6  3/112 |  |  |  |  |  |

The chromosome numbers in one nucleus (x) and in the second nucleus (y) within a cell are shown as x+y values. The grey-shaded column represents the most frequent karyotype variant (prevailing karyotype) for the respective WBc6-Cande passage. Other columns represent other karyotype variants found. The passage number (px) and the date of analysis are indicated in the first column for the WBc6-Cande passage. No prevailing karyotype change was observed in this *Giardia* line.
