# Supplementary material for: Constitutive aneuploidy and genomic instability in the single‐celled eukaryote Giardia intestinalis
Source: Microbiologyopen. 2016 Mar 23;5(4):560–74. doi: 10.1002/mbo3.351 (PMC4985590; doi:10.1002/mbo3.351)
Supplement: Supplementary file 6 — Table S3. The table shows the percentages of probe binding patterns in the two Giardia nuclei observed by single‐color FISH in interphase and mitotic nuclei. N indicates the number of cells, in which the binding pattern was evaluated. [file MBO3-5-560-s006.docx]

Table S3:

The table shows the percentages of probe binding patterns in the two *Giardia* nuclei observed by single-color FISH in interphase and mitotic nuclei. *N* indicates the number of cells, in which the binding pattern was evaluated.

| Probe | **Chromosomal binding patterns**  **observed by FISH (in %)** | | | | | | |
| --- | --- | --- | --- | --- | --- | --- | --- |
|  | **2+2** | **2+1** | **2+3** | **1+1** | **3+1** | **2+0** | **3+0** |
| ***rad***  n=101 | **60** | 22.5 | 11 | 6.5 | 0 | 0 | 0 |
| ***iso***  n=81 | **57.4** | 23.3 | 9.7 | 3.2 | 6.4 | 0 | 0 |
| ***ubi***  n=95 | **59** | 19.6 | 13.7 | 1.9 | 5.8 | 0 | 0 |
| ***tert***  n=105 | 15 | **45** | 9 | 14 | 5 | 8 | 4 |
