# Supplementary material for: Constitutive aneuploidy and genomic instability in the single‐celled eukaryote Giardia intestinalis
Source: Microbiologyopen. 2016 Mar 23;5(4):560–74. doi: 10.1002/mbo3.351 (PMC4985590; doi:10.1002/mbo3.351)
Supplement: Supplementary file 7 — Table S4. Outcomes of a bioinformatic search for orthologs of major tumor suppressor factors and checkpoint activators implied in the ploidy control. [file MBO3-5-560-s007.docx]

Table S4:

Outcomes of a bioinformatic search for orthologues of major tumour-suppressor-factors and checkpoint activators implied in the ploidy control. The table displays the sequential search for *Giardia* orthologs. *S. cerevisiae* and human protein sequences were used as queries for BLASTP in GiardiaDB database, consequently, the found giardial protein sequences were employed for reciprocal BLASTP. The hits resulting from the initial search were further compared to all accessible protein sequences in NCBI database and within Pfam database. Final proposal and corresponding references are indicated.

| ***S. cerevisiae*** | *BLAST against GiardiaDB (query: yeast AA sequence)* | *Reciproc.BLAST* | **Human** | *BLAST against GiardiaDB (query: human AA sequence)* | *Reciprocal BLAST* | *BLAST against NCBI (query: Giardia AA,*  *results excluding hypotetical proteins)* | *identified Pfam family (query: Giardia AA)* | ***Summary for Giardia*** |
| --- | --- | --- | --- | --- | --- | --- | --- | --- |
| absent |  |  | p53  AAC12971 | 1.8  hypotetical protein  (GL50803_95290) | cellular tumor antigen p53 isoform d | Protein Bm10793, isoform i [B. malayi]  p53 [E. baileyi] | not found | **absent**  (Rutkowski et al., 2010) |
| absent |  |  | pRb  P06400 | 2.5  Phospholipid-transporting  ATPase IA, putative GL50581_177 | 6e-63  ATPase, aminophospholipid transporter (APLT), class I, type 8A, member 1 variant [H. sapiens] | 1e-69 Aminophospholipid translocase (flippase) ... [Komagataella pastoris] | E1-E2 ATPase, haloacid dehalogenase-like hydrolase  domains | **absent** |
| absent |  |  | p21  AAB29246 | absent |  |  |  | **absent** |
| absent |  |  | p73  NP_005418 | 4.5  GL50803_27967 product=unspecified product | 2.7  tumor protein p73 isoform k [H. sapiens] | 3.0  F420-dependent protein [Mycobacterium sp. UM_RHS] | not found | **absent**  (Rutkowski et al., 2010) |
| Dbf2p  AKB01464 | 3e-61  **GL50803_8587**  Product: Kinase, AGC NDR | 2e-89  Cbk1p [Saccharomyces cerevisiae YJM627] | Lats2  Q9NRM7 | 8e-75  **GL50803_8587** \| organism=Giardia_Assemblage_A_isolate_WB \| product=Kinase, AGC NDR  and other kinases | 2e-74, serine/threonine-protein kinase LATS2 [H. sapiens] | 8e-104 Serine/threonine-protein kinase tricorner [G. arboreum] | Protein kinase domain | **GL50803_8587 Kinase, AGC NDR**  (Manning et al., 2011) |
| Mad3  CAY80709 | 5.1  GL50803 hypothetical  protein | 2e-04 Utp6p [S. cerevisiae FostersB] | BubR1  O60566 | 0.012  GL50803_9422 \| organism=Giardia_Assemblage_A_isolate_WB \| product=Kinase,  CMGC CDK | 3e-32, cyclin-dependent kinase 3, isoform CRA_a [H. sapiens] | 3e-31  p34-cdc2 protein [D. discoideum AX4] | Protein kinase domain | **absent**  (Manning et al., 2011) |
| HOG1 protein  AAA34680 | 9e-93  **GL50803_17563**  Product: Kinase, CMGC MAPK | 1e-102 Fus3p [S. cerevisiae YJM1574] | p38  syn. MAPK 14  NP_620583 | 1e-80  **GL50803_17563**  Product: Kinase, CMGC MAPK  and other MAPK kinases | 3e-102 Chain A, Crystal Structure Of Mapk7 (erk5) With Inhibitor [H. sapiens] | 5e-124 extracellular signal-regulated protein kinase [D. purpureum] | Protein kinase domain | **member of CMGC MAPK kinases family found**  (Manning et al., 2011) |
