# Supplementary material for: Constitutive aneuploidy and genomic instability in the single‐celled eukaryote Giardia intestinalis
Source: Microbiologyopen. 2016 Mar 23;5(4):560–74. doi: 10.1002/mbo3.351 (PMC4985590; doi:10.1002/mbo3.351)
Supplement: Supplementary file 8 — Table S5. Information regarding the probes used in FISH experiments on Giardia intestinalis chromosomes. [file MBO3-5-560-s008.docx]

| **gene** | **chromosome** | **gDB Accession Nr.** | **size (bp)** | **gene location on chromosome** | **forward**  **primer** | **reverse**  **primer** |
| --- | --- | --- | --- | --- | --- | --- |
| ***rad50*** | 4 | GL50803_17495 | 2001 | 100.005-104.168 | 5´-TGTGCTGCAAACAGCGATCA-3´ | 5´-AGACGTCGCAAGAAACTGCT-3´ |
| ***telomerase catalytic subunit (tert)*** | 4 | GL50803_16225 | 2079 | 703.969-706.851 | 5´-GCCTCCCTATGGTCACTTAT-3´ | 5´-GGACGAAACAATCCCTGTATTT-3´ |
| ***isoleucyl-tRNA synthetase* (*iso*)** | 4 | GL50803_104173 | 2150 | 2.465.185-2.468.682 | 5’-GCCTGTGCTACGCCTCTATC-3’ | 5’-AGCGCAGTCATCTTCTGGAT-3’ |
| ***ubiquitin* (*ubi*)** | 4 | GL50803_13701 | 2393 | 2.696.695-2.704.199 | 5’-GTTCGACAAGGACCTGGTGT-3’ | 5’-CCAAACTATCGTGCAGAGCA-3’ |
| ***actin* (*act*)** | 2 | GL50803_40817 | 1081 | 1.297.290-1.298.417 | 5´-TGATAACGGCTCCGGAATGT-3´ | 5´-TGCAATGGAAGCGCCATTCT-3´ |
| ***ser/thr phosphatase* (*ser*)** | 5 | GL50803_7439 | 1900 | 1.456.301-1.458.259 | 5´-CAAGGACACGGTCTTTCCTATT-3´ | 5´-GTCACTCGTCAGGTTCATCAG-3´ |

Table S5

Information regarding the probes used in FISH experiments on *Giardia intestinalis* chromosomes.
